# Supplementary material for: An electrophysiological and kinematic model of Paramecium, the “swimming neuron”
Source: PLoS Comput Biol. 2023 Feb 9;19(2):e1010899. doi: 10.1371/journal.pcbi.1010899 (PMC9946239; doi:10.1371/journal.pcbi.1010899)
Supplement: Attachment — Submitted filename: Response to reviewers.pdf [file pcbi.1010899.s008.pdf]

Response to reviewers

Dear editor and reviewers,

First of all, we thank the reviewers for their detailed comments and their appreciation of this work ("A methodologically and scientifically rigorous, ethically conducted, and objectively presented" (#1); "provides an interesting and broadly speaking successful example of this integration" (#2); "the paper is of high quality overall" (#3)).

The reviewers also pointed out some important limitations of this work, in particular the fact that the electrophysiological model is much more experimentally constrained and detailed than the modeling of motility. We generally agree with the reviewers, since as they noted, we pointed out those limitations explicitly in the manuscript ("The authors acknowledge the limitations of the model" (#1); "The authors are commendably upfront and clear about the limitations" (#3)).

Thus, we would like to clarify the aims of the paper and what we think we have achieved. Our goal was to develop an electrophysiological model of the action potential of *Paramecium*, and to connect it to motility so as to obtain an integrative model that may guide further investigations into the physiology of behavior of *Paramecium*. The closed-loop scenarios presented in Fig. 8 and 9 are not predictions of actual behavior meant to be tested directly – they cannot be since the stimulus is generic. Rather, they are illustrations of how the integrated model can be used for investigating the physiology of behavior. One must realize that in order to make a proper quantitative investigation of any kind of behavior, the model must be augmented with the physiological aspects of sensory transduction as well as the physical aspects of the stimulus, both of which represent substantial specific work. For example, modeling mechanosensitive behavior with obstacles requires modeling mechanical transduction as well as mechanical interaction between cell and obstacles (and fluid), both of which are difficult subjects. This was stated at the end of the discussion (l.872-873, "Thus, the model should be completed by models of sensory transduction, as well as of mechanical interaction with objects. This would allow us to use the model to investigate the physiological basis of behavior of *Paramecium* in complex environments"), but we have now inserted it earlier in the revised text, in the Results.

In that regard, we realize that the title of the paper may be misleading ("an electrophysiological and behavioral model"), since we do not model any specific behavior but rather the coupling of the action potential with motility. We propose to replace it with the following title: "An electrophysiological and kinematic model of *Paramecium*, the swimming neuron".

The reviewers are correct that the electrophysiological modeling is much more detailed than the coupling with motility. We concur that this is an important area for further work. Most of our experimental efforts have been focused on the electrophysiology, which we have then coupled to motility with a minimal phenomenological model. Since the reviewers may not be fully familiar with electrophysiological work, we would like to emphasize that developing an empirically constrained electrophysiological model of a cell is not trivial at all. Although it might not appear so in the paper, obtaining the experimental data represents considerable work, especially as *Paramecium* is a motile cell. The cell needs to be immobilized prior to the experiments (which we did by building a microfluidic device), and even then, it may still make movements (e.g. contractions). Measuring fluid motion simultaneously with electrophysiology is also technically difficult (as noted by reviewer #2), and comes with a number of limitations (see detailed responses below). In addition, multi-objective fitting of complex nonlinear differential models to different data types is not trivial (we actually developed a specific computational tool to do this). Finally, we would like to stress that building an electrophysiological model (or any model) does not simply consist in constraining parameters with data, but firstly in developing the formal model itself, based on a thorough analysis of the electrophysiological literature and of the quantitative constraints. To our knowledge, this is the first empirically constrained model of the action potential of a protist, and *a fortiori* the first model integrating electrophysiology and motility, despite its limitations.

To couple electrophysiology with motility, we simply postulated a direct modulation of the three main kinetic parameters by calcium concentration, as it is known that calcium controls ciliary reversal. The analysis of the spherical model was meant to provide a plausible way in which this modulation may arise, as well as to give some insight into the functional form of the calcium-kinematic couplings (but the spherical model is not included in the final model). Although this modeling represents substantial work, it is indeed loosely constrained by experimental data and we fully agree that it must be considered as more hypothetical than the electrophysiological model. In order to validate the calcium-kinematic couplings, one would first need to measure calcium concentration in the cilia. So far, we have not managed to do it, presumably because the cilia represent only 2-3% of the total volume of the cell. A technical solution would be to target a genetically encoded calcium sensor to the cilia. In addition, the measurements should be done in a freely swimming cell in which we would measure the trajectory in 6D (position and orientation) to extract the kinematic variables. Empirical evidence for the spherical model connecting ciliary heterogeneity to calcium-kinematic couplings would require spatially resolved calcium imaging together with high-speed imaging of cilia beating. All of these are technically possible but are full-size experimental projects on their own. Although it might be frustrating that not all parts of the integrative model have the same level of empirical grounding, we do not think it is reasonable to expect such a level of detail in a single paper (which is already 48 pages long), as long as the paper does not claim otherwise.

While these are certainly limitations of the model, the model provides plausible quantitative hypotheses and suggests specific ways to test them (as described above), which we think is one of the goals of modeling work.

We have tried to clarify the aims and limitations of the paper at different places. We also added a synthetic figure to summarize the model, as a guide for the reader. We hope that you will appreciate this revised version.

Best regards,
Romain Brette

Detailed responses
Note: in our responses, line and figure numbers refer to the revised version.

Reviewer #1 (Public Review):

As far as I can assess, the manuscript is methodologically and scientifically rigorous, ethically conducted, and objectively presented.

My main concern is about the level of novelty (i.e. about what new we actually learn from it). Although I am not myself an expert in electrophysiological data, I believe there is a major experimental and modelling effort by the authors to promote Paramecium tetraurelia as a model system for neuromechanical behavioural studies. The work leads to certain constrained values for the electrophysiological parameters, although under notable modelling constraints (see my comment below). This is laudable and will certainly be of interest to the community dealing with the "physiological basis of autonomous behaviour" in general, and electrophysiology in particular.

Once coupled to swimming kinematics, the "effective/phenomenological model" reproduces qualitatively observed behaviours in terms of cell interaction with a structured environment and/or cell-to-cell interaction. This is a valid endeavour but, in a way, the results of which are not totally unexpected: while the model may be extremely realistic and detailed, at the level presented in the manuscript it mainly describes mathematically the existing data, not necessarily imbuing it of predictive power to explore unknown aspects of the system. It is true that estimates from the electrophysiological model compare reasonably well with previous values obtained by different

means. But the resulting dynamics of the "swimming model" are not quantitatively compared with experiments on cell behaviour. The authors acknowledge the limitations of the model and specify a number of directions in which it can be expanded and/or improved. I would have loved to see more specific predictions emerging from the model and those being compared with "a posteriori" measured experimental data. To this referee that's an overall weakness of the approach.

Thank you for your comments. These general issues are addressed in the general response above. As explained, our goal in this paper was to build an integrated model, not to make any new claim about behavior or electrophysiology. That the model matches the data was our goal, so it is certainly not unexpected, but not trivial either. It was not at all obvious, for example, that the set of mechanisms inserted in the model (ionic channels and calcium pathways) would be sufficient to reproduce the data.

Where the model is less empirically constrained, as for the coupling between calcium and motility, it is also a way to explore possible hypotheses, check whether they might be able to underlie the phenomenon and suggest specific experiments. We believe this is the case for our proposed model of the coupling of electrophysiology and motility.

Reviewer #1 (Recommendations for the authors):

Minor comments follow:

- I couldn't find in the ms any experimentally measured baseline statistics for avoidance reactions in the absence of stimuli.

The rate of avoiding reactions in our behavioral measurements was 0.2 Hz (l. 665). Statistics for angle and backward swimming duration are given in Fig. 7H.

- Why was PIV performed in a plane 30um above the cell? what's the vortex-like flow? why is the flow in C1 described as moving "forward"? flow in the immobilised case has to be modified by the presence of the electrodes; I can't see that in the data; why?

The flows above and below the cell are in opposite directions, so we must image substantially above the cell to avoid conflicting motion cues. We cannot see the full flow here because the field of view is not large enough. In C1, the average flow points to the posterior side (and right) of the cell, so the cell would move forward (and spiraling left).
The electrodes are very thin (<1 µm) and tilted by about 30°, so they make only a small incursion in the focal plane.

- Why not just focus on an ROI for the PIV data? e.g. on the side of the cell. The flow structure makes it more accurate for measuring this response than looking at the cosine of the average field.; why didn't they look directly at cilia directional motion on different spots over the cell surface?

We did in fact try hard to get a more spatially resolved measurement of PIV, but it turned out to be impossible in many cases due to technical limitations. We found out that the mean angle (circular mean) of the vector field was the most robust measurement. Because we use a filter device with a pump to immobilize the cell, the particles must be inserted after the pump is stopped, which is when the electrodes are in place. The fluid volume is quite large and the depth is not uniform, because the cell cannot be covered with a coverslip (because of the electrodes). So the density of particles is not precisely reproducible and not stationary (sedimentation), while the electrophysiological recordings last for several minutes. Choosing a small ROI was generally possible only in a subset of frames. The best we managed to do reliably in a subset of recordings was to measure the average flow in the anterior and posterior sides (Fig. 6F).

Measuring cilia motion directly would be an option (although the optics is not optimal with the filter used for immobilizing the cell), but it requires a high-speed camera with excellent spatial resolution, which we do not have.
We added a comment in the discussion ("Limitations").

Reviewer #2 (Public Review):

The paper focusses on the link between physiology and motility in the common protozoan Paramecium. This species has been long studied in regards to both its bioelectric properties and its motility, but the two have not been linked together yet. The authors combine single-cell measurement of membrane potential, electrical stimulation, and measurement of (parts of) the flow field around the microorganism, and start by developing a mathematical model for its whole-cell action potential. This is the result of the dynamics of two main ions involved in modulating the transmembrane potential: potassium and calcium. In turn, intracellular calcium, which seems to be confined to the ciliary lumen, is also known to modulate the motility of cilia. The authors strive to quantify the effect of electrical stimulation on flagellar behaviour by measuring the flow field in a region around the microorganism through particle image velocimetry. The result is that a sufficiently intense stimulus leads qualitatively to a reversal of the flow field followed by a short swirling state before the cilia return to normal behaviour. Combining the action potential model with ciliary activity and assuming a heuristic model for the sensory transduction of environmental stimuli, the authors then develop a mathematical model for a swimming Paramecium which is capable of displaying a range of behavioural responses similar to the actual microorganism.

I find it difficult to decide whether the manuscript's conclusions are justified by the data because I am not completely sure what the claims of the authors are. In regards to their electrophysiological model's ability to recapitulate the experiments, this seems to me to be well justified. Regarding the dependence of motility on intracellular calcium concentration, I found the discussion quite difficult to follow. The fact that there is dependence is clear, but it is kept in the paper at a very qualitative level. The model for the effect of external stimuli on the action potential is an ansatz. Overall, the general structure of the interplay between membrane potential, motility, and environmental stimuli is there, but the implementation becomes more and more qualitative the more one advances through the paper. It is not clear to me whether this is a problem, because - as written above - it is not clear to me what the final claim of the paper is.

Thank you, we agree with your assessment. We hope that our general response above clarifies the matter. The goal of the paper is to build an integrative model, rather than to make any specific empirical claim. A result of the modeling work, however, is to propose specific testable hypotheses (rather than claims) that can be further investigated (for example about the control of turning by calcium).

Strengths
The underlying motivation of the paper is to link motile behaviour with intracellular physiology. This is an area that is currently still insufficiently explored. The manuscript provides an interesting and broadly speaking successful example of this integration within a context at the intersection between electrophysiology and microbial motility.
The authors present as well a detailed experimentally led and validated model of the action potential within Paramecium, which extends greatly what is currently available.
The integration of electrophysiology experiments with PIV of the fluid flow around the microorganism is novel, interesting, and potentially of wider applicability.

Thank you.

Weaknesses
1) The full model, including stimulus, action potential, and motility, is not really compared in any way

to the behaviour of the real microorganism. It is not clear to which extent, qualitatively and quantitatively, the behaviour of the model reproduces that of real Paramecia.

> *That is essentially correct. As explained above and mentioned in the discussion (Perspectives), a quantitative comparison to behavior would require augmenting the model with sensory transduction and physical aspects of stimuli (e.g. mechanical interactions). What can be said is that the model swims in spirals and does avoiding reactions that are qualitatively similar to real paramecia (backward swimming followed by reorientation with similar orders of magnitude; graded strength; and also reorientations without backward swimming), as stated in the Discussion (l. 762-764). Behaviors shown in Fig. 9 are mostly examples meant to demonstrate the use of the model, but they cannot be directly compared to behavior since there is no specific sensory modality. But Fig. 9A and 9B resemble attraction and repulsion by drops of various chemical substances as shown by Jennings (1906), paramecia swim along thermal gradients similar to Fig. 9C, and Fig. 9D qualitatively resembles the breathing-induced behavior described by Jennings.*

2) The coupling of motility with intracellular calcium concentration is only very loosely linked to the experiments. The general idea is reasonably in line with the data presented, but the details are not really strongly supported by experiments. The analysis of the PIV data to try and extract more detailed information is something that should be improved.

> *We agree. We consider it as a plausible hypothesis. The interest of the model here is that it shows that the hypothesis can account for a functional avoiding reaction (backward swimming then turning). But certainly, it would need to be tested with specific experiments (see general response). This is acknowledged in the discussion (Limitations and Perspectives).*

> *We also agree that the analysis of PIV data is a bit disappointing, but unfortunately this is due to the technical limitations of simultaneous electrophysiology and PIV measurement (see our response to reviewer #1). What is shown in the paper is the best we could make of our data. Possibly, direct measurement of ciliary beating could give us richer data.*

3) There is a striking contrast between the effort done for understanding and modelling the action potential, and the one done for the motility and stimulation. In my opinion, this lets the paper down.

> *That is also correct, but we do not claim otherwise. As explained in the general response, we have indeed focused here on the electrophysiology, while the coupling with motility is more phenomenological. We feel that the manuscript is already quite dense (cf next remark) and it seems more reasonable to leave this to further work.*

4) The manuscript is not an easy read. I often found it difficult to follow the main logical thread of the text.

> *The fact that the manuscript touches on different aspects of modeling (electrophysiology, hydrodynamics, kinematics, behavior) certainly makes it difficult and technical. We have added a synthetic figure at the beginning (Fig. 2) to summarize the known physiology and the modeling, and edited the manuscript at different places.*

Reviewer #2 (Recommendations for the authors):

Below I summarise the main suggestions I have for the authors.
A) I find the section which builds the model for the action potential interesting but quite difficult to follow (disclaimer: I am not an expert in electrophysiology). The system is clearly quite complex and in fact, the authors wanted to give an initial overview of what is known about its structure. In my opinion, this needs to be largely rewritten to specify in a clearer way what is known. An

accompanying sketch and circuit diagram would help greatly, as would the explicit definition of the different symbols within the main text.

Since the majority of the work is electrophysiological, we understand that this can be difficult, but we cannot dismiss the technical details. The electrophysiology of Paramecium has been reviewed in detail elsewhere (Brette, eNeuro 2021). We have followed your advice and added a synthetic figure (Fig. 2) to summarize the known physiology and the modeling.

B) The analysis of the PIV data needs to be improved and streamlined. It is not immediately clear at the moment why the authors look both at the average flow in the front/back of the cell (giving the cos(\alpha) curves), and at the average flow in the whole of the field of view. The former, in a narrower region, could already be used to estimate the difference between the thrusts from the front and back of the cell and how this would then translate to a whole cell rotation (e.g. by estimating the stress on the fluid necessary to generate that flow). This is in the direction of what is already done in Fig.5F, which looks at the direction of the average flow, which however does not seem to be used.

See our response to reviewer #1. We did in fact spend a lot of time on the analysis of the PIV data, but unfortunately this is the best that we could obtain in a reliable way.

C) I am a bit confused by the "double" modelling of the motility, which is not clearly explained in the main text. What is the reason for that? Is the spherical model used only to estimate the maximum spinning rate? The spinning rate is the result of complete guesses about the behaviour of the stress on the surface of the organism as well as of the assumed equivalence between the sphere and the cell body, which is not a given. Notice as well that for the spherical model with A the force per unit area (to be corrected in line 1120) and r the radius of the sphere, we have that U is proportional to Ar (A times r) whilst \Omega is proportional to A. This means that one can easily change \Omega while keeping U at 500um/s, by playing with A and r simultaneously. Therefore I do not see a compelling reason to give much quantitative weight to the 7.2Hz cited in the paper (line 383). Besides, the fact that the cell is not a sphere means that it cannot really be represented faithfully by one. So the idea of the swimming model should be, in my opinion, to provide something akin to a minimal model for this swimming neuron. I would not be at all against this approach, but then what is gained by doing the significantly more convoluted model in the section "Kinematics"? (and the confinement to a plane by constant re-rotation looks bizarre to me).

The spherical model is introduced only to understand qualitatively how ciliary reversals may lead to turning, and what the coupling between calcium concentration and kinematic variables may look like. But in the swimming model (Fig. 7), we only need to specify those couplings (shown in Fig. 7A-C).

In any case, we then need to translate the kinematic variables from the local coordinate system to the global coordinate system and integrate them in order to obtain trajectories. This is what is done in the section "Kinematics", which does not replace at all the spherical model. What replaces the spherical model is the phenomenological description of the couplings between calcium concentration and the three kinematic variables (Fig. 7A-C). Therefore, the model used in behavioral simulations is not more convoluted but on the contrary, it is a simplified version. The specific numbers (e.g. 7.2 Hz) shown for the spherical model are not used in the phenomenological model. Also, r is fixed (at 60 µm) to be roughly of the right order magnitude (Paramecium is 120 x 35 µm); but of course, we agree that the results are only qualitative anyway. We chose a sphere because analytical work is possible.

We tried to clarify these points in the text.

D) Finally, regarding the closed loop model: I find the whole idea of this model interesting, but I have to admit that I am not sure what have we learned from this. It seems to me that way the environmental stimulus is implemented is exactly done in order to elicit the type of behaviour we

want to see.

As explained in our general response, these behavioral scenarios are meant as illustrations of how the model can be used in further investigations, they are not meant as claims about specific behaviors. Specifically, the model can be used to explore plausible hypotheses and check whether they may be consistent with the observed phenomenon, what are the key components of the model and so on. For example, Fig. 9B shows one way in which a stimulus can be made attractive, i.e., a plausible hypothesis. The simulation also shows some properties that may or may not be realized in actual behavior (which will depend on the actual stimulus, e.g. the specific chemical substance), namely long backward swimming across the drop and a tendency to aggregate near the boundary, because of drop concavity. Thus, the model (as any model) offers a way to compare mechanistic hypotheses with observed behavior. But the aim of this paper is to develop the model, not to investigate a particular behavior, which would be an entire study by itself. We tried to clarify in the revised text.

Reviewer #3 (Public Review):

This careful paper models the transformation from environment to movement in paramecium, with a particular focus on the biophysical properties of the cell. The core framework puts together two model components: a detailed data-constrained biophysical model for the action potential, and a kinematic model for how movement is driven by cilia motion. While these pieces are quite mechanistically developed, multiple aspects of the transformation are phenomenological: namely how environmental stimuli are transformed to a voltage input and how Ca affects cilia movement. Parameters of a phenomenological model are partially constrained by experimental measurements. By assembling this sequence of steps, the paper describes simulations of several types of behavior by guessing how different inputs may be transformed into current signals driving the biophysical model. For example, in a simple closed loop, the model is used to show how movement can lead to different environmental conditions which in turn affect behavior. The model makes two predictions about the required inputs needed to drive two specific behaviors-namely that avoidance requires persistent stimulation, while attraction cannot be explained under the assumptions of the model without requiring that there is adaptation to a hyperpolarizing input. However, a key weakness of the paper is that in the transition to modeling behavior, there is little use of experimental data to pose questions or validate model components, so the status of these predictions is quite speculative. Thus while the paper is of high quality overall, this is a significant lack. The authors are commendably upfront and clear about the limitations in the chain of the derivation of model steps, but the work would benefit from additional context.

Thank you. As explained in our general response, these behavioral scenarios are meant as illustrations of how the model can be used in further investigations, they are not meant as claims (or predictions) about specific behaviors. We tried to clarify in the revised text.

Specific comments:

1. Comparisons of the model with behavior: the authors have assembled a quite large database of paramecium behavior. However, the modeling of this behavior seems somewhat anecdotal (ie. a single recorded example trajectory is shown in Fig 6E) relative to the potential quantification available from the recorded behavior. Is there more that one could do to extract something interesting from the behavior? For example, are there statistics of the movement that the model replicates? Despite the figure title, Fig. 6H is presumably taken from recordings. It is very unclear how to relate this data to model predictions.

Our behavioral data are only freely swimming trajectories, measured in 2D. Thus, there is not much more that can be done here than statistics of spiraling paths (swimming velocity, angle) and avoiding reactions (reported in Fig. 7H).

The comparison with the model is indeed qualitative. We simply show that the orders of magnitude for backward swimming duration and reorientation angle are reasonable, and that the two quantities are not lawfully related, although correlated. It is not possible with these data to go much further, because there is no stimulus. Or, more precisely, the spontaneous avoiding reactions are presumably driven by intrinsic noise, which is unknown.

2. How does the avoiding behavior of Fig 7 compare with real behavior? In the formation of aggregates, is there anything to compare with to assess how the model compares with data?

We cannot give a general answer to the first question, because it depends on the stimulus. Responses to light are slow, mechanical responses are fast. In our experience, the behavior shown in Fig. 8A is not observed with mechanical contact.
To our knowledge, the formation of aggregates has only been described by Jennings (more than a century ago). Therefore, at this stage, the comparison can be only qualitative. But our aim was simply to show that the model can exhibit this kind of collective phenomenon. A precise comparison with behavior would require a specific study.

3. Electromotor coupling: This step contains a simplification that steps over mechanistic issues to treat the cilial beating direction, velocity, axis angle, and spinning speed as each is driven through a Hill or bell function by calcium concentration. The relationship between these definitions and the kinematic model is a bit confusing. In the kinematic model, velocity and spin are derived from the forces that are established by the cilial angles defined over the surface of a sphere. These vary across the body, and indeed need to generate any movement (although the section on paramecium swimming suggests otherwise, presumably only the cilia on the side of the body opposite to the direction of motion really participate in a forward movement?). While in the kinematic model, the core descriptor of (spatially varying) cilial angle defines the forces which define the movement, it was unclear how these can simultaneously be established by the relations of this section-if the interrelationships between these functions are derived from the kinematic model, it would be helpful to reorganize to make that logic clear.

There might be a misunderstanding. The hydrodynamic model relates forces exerted by the cilia to kinematic variables, and is used as a way to explore a plausible relation between calcium concentration and those variables, as well as to substantiate a plausible hypothesis for the mechanism (asynchrony of ciliary reversal). The kinematic model does not explicitly use those forces, but directly uses postulated (phenomenological) relations between calcium concentration and kinematic variables.
We tried to clarify.

4. Calcium is the key dynamical variable, and is not directly measured experimentally; rather it is inferred by the measurement of the cilia angle, which is modeled as a Hill function of the Ca concentration. This is a key step that should be unpacked a little. Is it possible that the inferred novel properties of the calcium channels (4 binding sites) could be due to simplifications in the Ca-cilia transformation? Further, as the authors note there must be some spatial variation in the relationship between Ca and cilia: how might this single control variable be turned into a spatial one?

It would be indeed ideal to measure ciliary calcium directly, but so far, we have not managed to do it. It is indeed inferred through the model. This is discussed in section "Limitations" of the discussion, l. 785-791 (note that calcium is constrained by several processes: ciliary reversal, calcium current inactivation, calcium-activated potassium channels, calcium dynamics).

Regarding the number of binding sites, it turned out to be impossible to correctly fit the action potentials with a single binding site for inactivation, even without ciliary data. A high exponent is required to strongly shut down the current after activation. But this number is just as phenomenological as in the Hodgkin-Huxley model (sodium channel activation has an exponent 3, but the sodium channel has four repeat domains). Even though we think the high exponent is

due to the properties of the calcium current (rather than artifacts of the optimization process), the molecular structure may be more complex than suggested by this simple model.

The last question is addressed in the discussion ("How Paramecium turns", l. 842-847): "Physiologically, asynchronous ciliary reversal can be due to differential calcium sensitivity, that is, the calcium threshold for reversal might vary across cilia. This is the implicit assumption of our model. It could also be that there are differences in calcium entry or removal across cilia (e.g. a gradient of calcium channel expression, or calcium pumps, or calcium buffering molecules).".

Reviewer #3 (Recommendations for the authors):

1. Overall, rhetorically, it would help the reader to state what is the big picture goal of the work: aside from the general goal of a model from input to output, and the successful building of a more accurate model of the channel dynamics underlying the action potential, what specific mystery or question has been addressed by the work? Is data used to simply constrain the modeling and suggest dynamics, or are specific aspects of measured behavior targets for an explanation?

As clarified in our general response, the aim of paper is firstly to develop a model. We think that this model will be very useful for investigating the relation between physiology and behavior, but this paper does not target any particular behavior. We tried to clarify this point in the revised text.

2. Description and figure of paramecium swimming: as most readers will not know this literature, it would be helpful to clarify this section. For example, one should show a diagram of the organism clearly showing cilia all the way around the body and not just in a tuft as shown in Fig 5D. Figure 5F and the accompanying MS text and caption could be greatly improved to make clearer what has been measured and how these conclusions are drawn.

We added a panel showing the ciliature of Paramecium (Fig. 6E).

3. Figure 6F shows a difference in turning behaviors in response to a step current depending on the angle relative to the oral groove. Is this an observed behavior? The kinematic model gives no special significance to specific locations on the body.

This is indeed described by Jennings. One simple aspect is that, when the organism moves in 3D while we observe it in a fixed plane, the direction of the movement relative to the observation plane will depend on the initial orientation of the organism in that plane. Hence, the organism appears to randomly turn left and right when it is in fact always doing the same movement, but spinning between consecutive avoiding reactions (described as "pseudo-random" in Brette, eNeuro 2021). Another aspect is that in Fig. 7, the movement is constrained to a plane, and so the movement will depend on the position of the oral groove relative to that plane.
We added some comments.

4. The biophysical modeling is carried out in a very thorough way. However, some additional clarity on the logic of its focus would be appreciated. The results start by modeling responses to hyperpolarizing pulses, and neglect the small Ca current (driven by hyperpolarization) in favour of fitting parameters of the inward rectifier, but then state that this will also be neglected because the study will subsequently focus on depolarizing pulses. It is a little unclear why the small calcium current, which mediates escape behavior, is neglected, as it evidently forms part of the behavioral repertoire. What kinds of inputs would differentially drive the inward rectifier current vs the small Ca current?

We chose to only model the avoiding reaction, not the escape reaction, which is why we did not consider the hyperpolarization-activated calcium current. It would be feasible, but we think this paper is already sufficiently dense.

The reason why we fitted the inward rectifier is because it gives us an indirect way to estimate the reversal potential of K^+, because that current opens below E_K. Once E_K is estimated, it is not necessary to include that current, since it is only active when the cell is hyperpolarized anyway.

We tried to clarify.

5. In principle the work allows one to reverse engineer the voltage signals that the environment must cause in order to account for specific behaviors, as the authors have suggested with respect to two behaviors - however, these seem underconstrained. For example, couldn't adaptation arise from the biophysical model rather than from sensory transduction? How would one fill in the gap in this step of the model? Is it possible to record current signals while externally changing environmental conditions in order to validate the model, or has this been previously undertaken?

Absolutely. The behavioral scenarios are meant as illustrations of how the model can be used, precisely to explore different hypotheses. We chose a particular one for simplicity. But indeed, excitability can also adapt. It has different properties, since the model we chose is stimulus-specific while excitability adaptation is unspecific (so it could be tested behaviorally). There is some electrophysiological literature that indeed finds changes in calcium current properties with prior voltage changes. To measure the adaptation of the sensory current is possible, one needs to measure the current in voltage-clamp while applying a step stimulus (e.g. thermal change, or chemical concentration change, etc). Of course, this requires specific additional investigations. We added a remark.

6. Minor point: it may be an exaggeration to call the model "hydrodynamic" rather than simply kinematic as there is no specific treatment of fluid properties.

We took the starting point from Lauga & Powers (2009), "The hydrodynamics of swimming organisms". Fluid properties are implicitly taken into account in the fact that motion vectors depend linearly on force and torque, which is due to the fact that motion operates at low Reynolds number. We wanted to distinguish it from the specifically kinematic treatment in the next section, where we simulate trajectories by integration of kinematic variables.
