# Supplementary material for: An electrophysiological and kinematic model of Paramecium, the “swimming neuron”
Source: PLoS Comput Biol. 2023 Feb 9;19(2):e1010899. doi: 10.1371/journal.pcbi.1010899 (PMC9946239; doi:10.1371/journal.pcbi.1010899)
Supplement: Attachment — Submitted filename: Review for PCOMPBIOL-D-22-01305.pdf [file pcbi.1010899.s009.pdf]

Review for PCOMPBIOL-D-22-01305

Summary:
First of all, the paper contains great science and the level of understanding of the model system is impressive. I think the paper is a major contribution to the field of computational neuroscience, moving from purely mechanistic models of neural activity to the neural basis of behaviour. The work provides many testable predictions, and I don't think it is problematic that the kinematic model is much less constrained than the electrophysiological model (as mentioned by previous reviewers), for two reasons. First, the model assumptions for the kinematic part are not wild guesses, but based on experimental data and a simple toy model with analytical results. Second, it is clear that in a single paper, it is impossible to model 'everything', and already the part dealing with electrophysiology merits publication on its own. In my eyes, it is ingenious to infer calcium-dependent properties from PIV measurements of ciliary reversal. I also think that the amount of experimental literature taken into account by the authors, some more than a century old, is impressive and shows how careful this study was conducted.

Thus, I fully support the authors in their claim that after devising a setup for electrophysiology and PIV, validating a model on the recorded data, and then deriving an experimentally motivated model for behaviour, it is out of scope for the current paper to compare the results to detailed behavioural experiments and add more detailed mechanisms for e.g. sensory transduction to the model.

Apart from modelling Paramecium, I think the data-driven, yet quite phenomenological approach could also be applied to other species with cilia, such as ctenophores (comb jellies) with multiple comb rows and complex nervous system structure.

That said, the paper is quite dense, and the general outline is hard to follow upon initial exposure to the content. This is mainly because many details are provided in an anecdotal, scattered fashion throughout the paper, instead of being provided in a concise broad overview at the beginning of the paper.

In my eyes, the paper could be improved if more details were moved to the Materials and Methods section because I was pretty overwhelmed with the amount of detail presented. I provide some suggestions for this below in Minor comments.
I then only have three major comments, which should be addressed by the authors.

Major comments:

1. Nomenclature for direction of currents: looking at the equation for the transmembrane voltage on l.907, I>0 (I<0) denotes a depolarising (hyperpolarizing) current. Is there any reason why I_K(Ca) is called 'outward' current, whereas I_Kir is called 'inward rectifier current',

even if both currents are usually hyperpolarizing (unless V<E_K for I_Kir)? On ll.157-163, it states that I_Kir switches from inward to outward- this is a bit confusing. Does it simply mean that the current switches from depolarising to hyperpolarising when the membrane voltage passes E_K from below? Please clarify.

2. Related to this, I_Ca in Fig.5F has a negative sign, and should therefore be hyperpolarizing. This is in contradiction to what I_Ca is supposed to do (it enters the voltage equation on l.907 with a positive sign and causes the depolarisation leading to the Paramecium action potential), which I find majorly confusing. Please clarify the convention used for the transmembrane currents. Thanks!
3. To make the fitting procedure more accessible, the authors should consider to add a schematic flow diagram depicting the main steps of the analysis and therefore exposing the logic of the whole approach. It could start with 1) the determination of E_K, then 2) the passive properties of the cell, then 3) determination of the current I_Kd using deciliated cells, and finallly end with 4), the fitting procedure of the whole action potential using PIV measurements. The main flow of information is from 1) to 4), but sometimes, there also is information flowing backward, e.g. when different mathematical formulations for the delayed rectifier current I_Kd are considered for better fits. Additionally, the electromotor coupling could be added as a last step to the flow diagram.

Minor comments:

1. General comment: it could be advantageous to summarise experimental knowledge on Paramecium (quantitative results on electrophysiology and swimming kinematics) in a single section and then refer back to that section when the model is derived in order to compare measurements and model predictions. Currently, the introduction is fairly general, and quantitative experimental results are mentioned along the way as the model is developed, which makes the paper harder to read.
2. l.54: It is a little bit unclear how exactly the approximately 4000 cilia are distributed on the surface of the animal. It only becomes clearer later in the paper (in Fig.6E). Maybe a small sketch showing relevant anatomical structures of Paramecium could be helpful here.
3. It is a bit confusing that the numbers in Fig.1A don't correspond to the numbers in Fig. 1 B and C
4. What is special about the position of the black arrow in Fig.1B? There does not seem to be a particular feature of the trace at this point. Similarly in Fig.5C.
5. l.193: Please clarify link between E_K and internal potassium concentration (GHK equations).
6. l.269 ff.: I am not sure that the whole section comparing the final

results to those of deciliated cells is well placed here. It is expected that the responses of ciliated and deciliated cells are different. Maybe it is only the first sentence of this section that is confusing, because the section does not compare deciliated vs. ciliated responses, but describes the action potential of normal, ciliated cells in general.

7. l.272: Please define the capacitive current.
8. l.277: I find the computation of the intracellular Ca concentration due to the current hard to follow, maybe just give the result without the equation and explain more in the Materials and Methods section?
9. l. 293: '(Leaving its parameters unconstrained)'- This is not clear to me- which parameters are unconstrained? Certainly not all of them.
10. Similarly, on l.298 ff., there is too much detail about modelling choices for the Ca current- some of which could be moved to the Materials and Methods section.
11. General comment for Fig.5 and its description: it is sometimes hard to discern what is experimentally measured and what is a model fit. Maybe some extra annotations in the plots would help.
12. Fig. 5A: I can't see the negative currents, presumably because they are much smaller than the positive ones.
13. l.317 (related to my major comment about the sign of currents above): I_{Ca} peaks at around -3.5nA, not at +3.5nA, as stated. Please make this consistent.
14. Typo on l.324: action potential is shown in Fig. 5C, not 5F.
15. Fig.6D: I am not sure what we are seeing here. Presumably 4 animals? Or 4 cilia? Please clarify. Also, the notion of 'oral groove' appears for the first time here- maybe a schematic anatomical drawing of a Paramecium at the beginning of the paper would be good (see minor comment 2 above).
16. General comment about omega: it took me some time to appreciate that Paramecium spins around its long axis while also moving forward according to the vector v. Maybe making this more explicit would be helpful for the reader.
17. L.343: It is not immediately clear what $\log_{10}(K_{Motor}$ in M) means and why it is mentioned, maybe this could be removed and the value, instead of its decadic logarithm, could be directly provided? Similarly for K_{Ca} on l.331 f.
18. Figure 8: How exactly is the 'cell area within the stimulus area' determined? This area increases when the cell moves into the stimulus region, and decreases when it moves out of the stimulus region, so it is not clear to me how to unambiguously define it once and for all (also not after reading the relevant section in Materials and Methods).
