# Supplementary material for: An electrophysiological and kinematic model of Paramecium, the “swimming neuron”
Source: PLoS Comput Biol. 2023 Feb 9;19(2):e1010899. doi: 10.1371/journal.pcbi.1010899 (PMC9946239; doi:10.1371/journal.pcbi.1010899)
Supplement: Attachment — Submitted filename: Response to reviewers.pdf [file pcbi.1010899.s010.pdf]

Response to reviewers

Dear editor and reviewers,

We thank the reviewers for their appreciation of our manuscript and their detailed feedback.

In the revised manuscript, we have inserted two new figures that serve as illustrations to improve readability. Figure 1 shows an image of *Paramecium* with its cilia and oral groove, a typical spiraling trajectory and a few typical behaviors. Figure 4 outlines the modeling strategy of the paper, referring to each individual figure and showing the connections between the different parts of the work. We hope this will help the reader navigate through the paper, which we acknowledge is dense.

Regarding the balance between Methods and Results, reviewer #1 suggested to move more details from the results to the methods, but reviewer #3 considered that too many details are found only in the methods. Therefore, we did not alter the balance. Perhaps there is no straightforward trade-off.

We edited the manuscript in various places to clarify the points highlighted by the reviewers. We also addressed all points raised by the reviewers (see Detailed responses).

We hope you will appreciate this revised version.

Best regards,
Romain Brette

Detailed responses
Note: in our responses, line and figure numbers refer to the revised version.

Reviewer #1

Summary:

First of all, the paper contains great science and the level of understanding of the model system is impressive. I think the paper is a major contribution to the field of computational neuroscience, moving from purely mechanistic models of neural activity to the neural basis of behaviour. The work provides many testable predictions, and I don't think it is problematic that the kinematic model is much less constrained than the electrophysiological model (as mentioned by previous reviewers), for two reasons. First, the model assumptions for the kinematic part are not wild guesses, but based on experimental data and a simple toy model with analytical results. Second, it is clear that in a single paper, it is impossible to model 'everything', and already the part dealing with electrophysiology merits publication on its own. In my eyes, it is ingenious to infer calcium-dependent properties from PIV measurements of ciliary reversal. I also think that the amount of experimental literature taken into account by the authors, some more than a century old, is impressive and shows how careful this study was conducted.

Thus, I fully support the authors in their claim that after devising a setup for electrophysiology and PIV, validating a model on the recorded data, and then deriving an experimentally motivated model for behaviour, it is out of scope for the current paper to compare the results to detailed behavioural experiments and add more detailed mechanisms for e.g. sensory transduction to the model.

Apart from modelling Paramecium, I think the data-driven, yet quite phenomenological approach could also be applied to other species with cilia, such as ctenophores (comb jellies) with multiple comb rows and complex nervous system structure.

Thank you very much for this positive assessment.

That said, the paper is quite dense, and the general outline is hard to follow upon initial exposure to the content. This is mainly because many details are provided in an anecdotal, scattered fashion

throughout the paper, instead of being provided in a concise broad overview at the beginning of the paper.

In my eyes, the paper could be improved if more details were moved to the Materials and Methods section because I was pretty overwhelmed with the amount of detail presented. I provide some suggestions for this below in Minor comments.

> *We have tried to edit the manuscript accordingly, in particular by inserting two new figures that serve as illustrations (Fig. 1 and Fig. 4).*

I then only have three major comments, which should be addressed by the authors.

Major comments:

1. Nomenclature for direction of currents: looking at the equation for the transmembrane voltage on l.907, I>0 (I<0) denotes a depolarising (hyperpolarizing) current. Is there any reason why I_K(Ca) is called 'outward' current, whereas I_Kir is called 'inward rectifier current', even if both currents are usually hyperpolarizing (unless V<E_K for I_Kir)? On ll.157-163, it states that I_Kir switches from inward to outward- this is a bit confusing. Does it simply mean that the current switches from depolarising to hyperpolarising when the membrane voltage passes E_K from below? Please clarify.

   The standard terminology for I_{Kir} is indeed "inward rectifier current", which might be a bit confusing given that K^+ currents are outward in the physiological range. The reason is that it passes current mainly when $V<E_K$, i.e., when the current is inward. Regarding the switch from inward to outward, yes this is indeed simply that V passes E_K from below. We tried to clarify (l. 201).

2. Related to this, I_Ca in Fig.5F has a negative sign, and should therefore be hyperpolarizing. This is in contradiction to what I_Ca is supposed to do (it enters the voltage equation on l.907 with a positive sign and causes the depolarisation leading to the Paramecium action potential), which I find majorly confusing. Please clarify the convention used for the transmembrane currents. Thanks!

   We agree it is confusing. The standard convention when showing ionic currents in electrophysiology papers is that I<0 is inward. This is the contrary of the usual modeling convention, which is I>0 inward (although sometimes the converse is used). The reason for the electrophysiological convention is that it is the current reported by the amplifier in voltage clamp, which is the current that needs to be injected so as to maintain the potential at a given value, and which is therefore the opposite of the membrane current. We wanted to show the traces with the electrophysiological convention so that they can be compared to previous voltage-clamp studies. We tried to clarify (l. 350-352 and caption of Fig. 7).

3. To make the fitting procedure more accessible, the authors should consider to add a schematic flow diagram depicting the main steps of the analysis and therefore exposing the logic of the whole approach. It could start with 1) the determination of E_K, then 2) the passive properties of the cell, then 3) determination of the current I_Kd using deciliated cells, and finallly end with 4), the fitting procedure of the whole action potential using PIV measurements. The main flow of information is from 1) to 4), but sometimes, there also is information flowing backward, e.g. when different mathematical formulations for the delayed rectifier current I_Kd are considered for better fits. Additionally, the electromotor coupling could be added as a last step to the flow diagram.

   Thank you for the suggestion. We have added a summary figure (Fig. 4), which outlines all the steps in the modeling strategy.

Minor comments:

1. General comment: it could be advantageous to summarise experimental knowledge on Paramecium (quantitative results on electrophysiology and swimming kinematics) in a single section and then refer back to that section when the model is derived in order to compare

measurements and model predictions. Currently, the introduction is fairly general, and quantitative experimental results are mentioned along the way as the model is developed, which makes the paper harder to read.

We added some of this information in the introductory section. However, some of it is difficult to present without entering into the detailed properties of the currents, which are introduced with the modeling.

2. l.54: It is a little bit unclear how exactly the approximately 4000 cilia are distributed on the surface of the animal. It only becomes clearer later in the paper (in Fig.6E). Maybe a small sketch showing relevant anatomical structures of Paramecium could be helpful here.

   We added an introduction figure (Fig. 1) with pictures of Paramecium.

3. It is a bit confusing that the numbers in Fig.1A don't correspond to the numbers in Fig. 1 B and C

   They actually more or less match (with the caveat that these are two different experiments, one freely swimming, the other immobilized). (1) is forward swimming, before the stimulus, (2) is the beginning of backward swimming, during the stimulus, (3) is during the turn.

4. What is special about the position of the black arrow in Fig.1B? There does not seem to be a particular feature of the trace at this point. Similarly in Fig.5C.

   There is a small upward inflection at this point, due to the calcium current. It is not very obvious because there is also the direct influence of the pulse stimulus at that time.

5. l.193: Please clarify link between E_K and internal potassium concentration (GHK equations).

   This is just the Nernst equilibrium. We added this mention (l. 249).

6. l.269 ff.: I am not sure that the whole section comparing the final results to those of deciliated cells is well placed here. It is expected that the responses of ciliated and deciliated cells are different. Maybe it is only the first sentence of this section that is confusing, because the section does not compare deciliated vs. ciliated responses, but describes the action potential of normal, ciliated cells in general.

   We edited the first sentence (l. 346).

7. l.272: Please define the capacitive current.

   Done (l. 348).

8. l.277: I find the computation of the intracellular Ca concentration due to the current hard to follow, maybe just give the result without the equation and explain more in the Materials and Methods section?

   We simply removed it, as it is mostly illustrative.

9. l. 293: '(Leaving its parameters unconstrained)'- This is not clear to me- which parameters are unconstrained? Certainly not all of them.

   Once the model is simplified, all parameters are unconstrained, except E_K (so these are just 4 parameters).

10. Similarly, on l.298 ff., there is too much detail about modelling choices for the Ca current- some of which could be moved to the Materials and Methods section.

We removed the calculation of the calcium flux at rest – this was only to motivate the introduction of the pump.

11. General comment for Fig.5 and its description: it is sometimes hard to discern what is experimentally measured and what is a model fit. Maybe some extra annotations in the plots would help.

    We edited the figure (now Fig. 7).

12. Fig. 5A: I can't see the negative currents, presumably because they are much smaller than the positive ones.

    Indeed, those are not shown, for clarity (otherwise panel E3 would be hard to read). They are not very important to show as those are essentially passive responses. We edited the caption (now Fig. 7A).

13. l.317 (related to my major comment about the sign of currents above): I_Ca peaks at around -3.5nA, not at +3.5nA, as stated. Please make this consistent.

    We edited (l. 418).

14. Typo on l.324: action potential is shown in Fig. 5C, not 5F.

    Indeed, thank you.

15. Fig.6D: I am not sure what we are seeing here. Presumably 4 animals? Or 4 cilia? Please clarify. Also, the notion of 'oral groove' appears for the first time here- maybe a schematic anatomical drawing of a Paramecium at the beginning of the paper would be good (see minor comment 2 above).

    It is a cell at different times during the rotation. We added an introductory figure (Fig. 1), with the oral groove.

16. General comment about omega: it took me some time to appreciate that Paramecium spins around its long axis while also moving forward according to the vector v. Maybe making this more explicit would be helpful for the reader.

    We inserted an explicit mention (l. 497).

17. L.343: It is not immediately clear what log_10(K_Motor in M) means and why it is mentioned, maybe this could be removed and the value, instead of its decadic logarithm, could be directly provided? Similarly for K_Ca on l.331 f.

    It is provided just before (2.4 µM). The logarithm is given so as to provide also the standard deviation (the optimization is done for the logarithm of K_{Motor}). We added the value also for KCa (63 µM) (l. 442).

18. Figure 8: How exactly is the 'cell area within the stimulus area' determined? This area increases when the cell moves into the stimulus region, and decreases when it moves out of the stimulus region, so it is not clear to me how to unambiguously define it once and for all (also not after reading the relevant section in Materials and Methods).

    This is in section "Sensory transduction" of the methods: at any given moment, we simply calculate the proportion of the cell shape that intersects the half-plane. This is indeed a time-varying current, which increases as the cell moves into the half-plane, then decreases (as shown on Fig. 10B, current I).

Reviewer #2

Summary

This work presents a complete biophysical characterisation of the action potential of the ciliate paramecium - once a key model for electrophysiology and behaviour and since forgotten. A major strength of the paper is its ability to discriminate between and measure (or infer) different kinetic parameters and ionic fluxes, such as through the use of a deciliation protocol. These carefully derived parameters are then used as input to a idealised swimming model to understand at a conceptual level the relationship between the membrane potential, ciliary orientation, asymmetry, and swimming trajectories. These integrative insights are supported in part by data, and extended to assess how the organism should respond to certain idealised environmental stimuli. The authors have also chosen to make public the codes and data associated with the paper which will make this a valuable contribution to the field.

This paper is very dense and contains a lot of information, this makes it a very hard read for anyone who is not familiar with the subject. It is difficult to keep track of the main message of the paper - the authors should consider simplifying some sections or removing some descriptions that do not necessarily add to the main narrative. The level of detail and rigour applied to the electrophysiological characterisation is not matched by the later sections on behaviour - this imbalance itself is due to many experimental limitations (which is fine to acknowledge) but can be addressed by structuring the paper in a more streamlined way.

For example the summary/discussions can be shortened, e.g. the section how paramecium turns appears to repeat some of the material from the results section. Descriptions of how specific model parameters lead to certain behaviours can be shortened/simplified - since there is probably over-interpretation of the model in some cases. In the case of the artificial scenarios considered, though these are interesting, the assumptions and equations behind them are not explained as carefully as the earlier sections of the paper.

> *Thank you, we tried to clarify the manuscript.*

1. Some structural issues:

It is also sometimes unclear which results/panels are from theory and which from experiments. Similarly, sometimes descriptions of results from previous work from other authors are merged with the current findings in the same paragraph (e.g. page 4 - 'when calcium enters cilia,…'). If the journal format allows, the authors should consider restructuring parts of the paper so it's more obvious which sections are results arising directly from their work and what is background info.

> *The section "A brief overview…" is indeed nothing new, we simply illustrate known facts with our own measurements (Fig. 1), which is a way to also introduce the techniques we used. We inserted an introductory sentence to make this more explicit. We also added "model" and "experiment" on the relevant panels.*

Another point is that since it was not possible to measure calcium concentration directly, a lot of references to 'x happens when calcium increases/decreases' are not supported by experimental data but are rather inferred, but the text sometimes makes this distinction ambiguous.

> *This concerns mainly the section on fitting responses of ciliated cells. We edited to make it more explicit (p 13).*

2. In general the figure captions are extremely long - can theses be shortened? Some of the information is repeated in the text.

> *They are quite long, but we think the captions should make all the notations explicit and the figures essentially understandable.*

3. The distinction between 'escape reaction' and 'avoidance response' is unclear, and the terms have

been used interchangeably in other species - though the authors are careful to state that they are only dealing with the latter. Then how is the escape reaction different from the avoidance response in terms of behaviour and electrophysiological properties, what actually happens in the paramecium escape, this should be made clearer or include the relevant citations.

This is on l. 203: "an increase in swimming speed triggered by hyperpolarizing stimuli". The citations were on l. 821, but we have now added them at this point.

4. In order to aid readability - perhaps there should be a figure panel summarising the idealised hydrodynamic model/whole-body approach? Perhaps one of the later figures should be reconfigured to make clear what is the role of the model and where it enters as part of the 'integrative model' - at the moment it's hidden deep in the supplement.

We inserted a figure summarizing the entire modeling approach (Fig. 4).

5. In general the PIV data was much more noisy than the ephys signal, and also is this enough to justify the claim that the cilia beat direction reverses longer than the corresponding potential signal? Not sure if scales with the signal intensity either - look at the purple and red lines?

Yes, PIV data can be quite noisy. It depends on the recordings, specifically on the density of particles when the video is made. The data are very clear on Fig. 8G. Nonetheless, even on Fig. 7B, all curves appear to be ordered by intensity except for the purple one.

6. Given that kinetic parameters are matched so precisely in the early sections, why is it that 'we simply set both the forward and backward maximum velocity at v = +/- 500 um/s?' (Bottom of page 15). The behaviour sections seems to be treated less rigorously than the action potential modelling - is there good justification for this?

In the previous section, we gave exact results of calculations in the spherical model. Here we chose values for the calcium-kinematic couplings for the integrated model, but since our behavioral measurements are less precise than the electrophysiology, we thought it would have been misleading to use values with several significant digits. Thus, velocity is 500 µm/s, spinning speed is 1 Hz, maximum angle is 90°.

The behavior section is not treated less rigorously, we simply do not have as much data to constrain the model. In particular, as explained in the previous revision, we would need 6D measurements (position + angles) of avoiding reactions at high frame rate, ideally with simultaneous calcium imaging, which we do not have at this point. Therefore, we made the best we could with the available information.

7. Some results according to Jennings or other older literature are referenced when it might have been possible to verify this from the new data, e.g. fig 6D - regarding the directional change in theta during the avoidance reaction, was this observed by the authors as well? What kind of stimulus method did Jennings use to get the different levels of avoidance response - and how does this relate to the injected current in the present study?

Our data are in freely swimming cells, there is no controlled stimulus, and the magnification is not sufficient to clearly see the three-dimensional movement and the oral groove during an avoiding reaction. Jennings used various kinds of stimuli, in particular chemical stimuli. He did not do electrophysiology, but he could study the effect of variations in chemical concentration. Thus, we expect only a qualitative relation with our injected currents.

8. One consequence of this interpretation is that the paramecium is very sensitive to the position of the oral grove at the onset of the stimulus, does this make sense physiologically and was this observed in the experiments? Or could this be an artefact of the fact that the movement is constrained in the plane in the simulations? This makes trying to infer any relationship between backward swimming and reorientation angle quite difficult from 2D projections of the complete 3D movement.

To be clear, Paramecium itself is not sensitive to the position of its oral groove, it is the behavior related to the environment or to the observation point that is sensitive to the oral groove position. Jennings writes that Paramecium always turns away from the oral groove, and this is also what we see (when we can see the oral groove clearly; it is not so easy to quantify it precisely without 3D measurements). Thus, as Paramecium continuously spins around its main axis, it will be seen to turn sometimes left, sometimes right, depending on where the oral groove is at that moment.

9. Clarify difference between open and closed-loop behaviour Why is chemotaxis considered closed loop…? This will be helpful for non-experts.

We clarified at the beginning of that section (l. 701-702). Chemotaxis is closed loop since the behavior of the organism has an effect on its future sensory input, which then impact its future behavior.

10. Some later section on CO2 and formation of aggregations - this is not well-explained, more details should be given either in the supplement and make some clear reference to where to find this information in the text. Page 21 line 584 and also later in that section - 'breathing' is the wrong word here, maybe respiration? Breathing is normally reserved for organisms with lungs and a full respiratory system? Also in the adaptation section - two pathways with opposite polarity were assumed, but it's unclear why? And also where the chosen time constants come from?

The technical details of the simulation with CO2 are given in the very last section of the Methods ("Collective behavior"), we edited it and inserted a reference in the Results (l. 800).

Indeed, "respiration" seems more appropriate, we edited.

Regarding the two pathways for adaptation, this is just the simplest modeling choice. Another possibility with a similar phenomenology would be an adapting current. Since the stimulus is generic here (it could be chemical or thermal, for example), we did not refer to any specific physiologically measured constant. This is meant to be an illustration of the kinds of behavior that the model can display.

Other queries

Figure 1C - what's the difference between the black and red arrows? Also in the caption - speaks of calcium current, but how do we know this?

The red arrows simply highlight the area neighboring the cell. We added this mention in the revised text.

The arrow in panel B points at a slight upward inflection in the voltage response. This implies that there is an inward current at this time. We know that it is a calcium current from the literature (in fact, in the present case, the extracellular solution only contains calcium and potassium, and only calcium currents are inward at this potential).

Fig 4d - box plot data has a strange distribution gkd - what's the reason for this?

It is just that there is an outlier with large conductance, otherwise it is not particularly strange. The reason for the outlier is probably that if the channel is not saturated by the stimulation (i.e., depolarizations larger than V_{Kd}), then there is an indeterminacy between V_{Kd} and g_{Kd}, that is, shifting V_{Kd} by k_{Kd} is equivalent to reducing g_{Kd} by a factor e. This explains also the outliers for V_{Kd}.

Fig 5 - missing a colorbar showing which I (nA) values correspond to which lines.

This is shown in A, bottom graph.

Fig 6 - how does one define anterior and posterior from the videos? Is this done roughly by eye or is the orientation of the cell accounted for by some sort of segmentation algorithm?

In Panel E - the labels should be defined, or perhaps remove any labels to structures that are not relevant.

This is explained in the Methods, section Particle image velocimetry (l. 1062-1068). It was done manually (by clicking on the two ends), then fixed based on fluid movement.

Panel E is not our figure, so we cannot easily remove the labels. We added the definitions in the caption.

Fig 7- can the layout of the panels be rearranged to make it clearer which label refers to which panel, e.g. F, G are a bit confusing. And why does panel G not have the equivalent of the red line (different orientation of the oral groove) in panel F? Also where is the oral groove in the simulations/model - can this position be highlighted? How exactly is the reorientation angle defined in both sims and experiments? If in panel H the same quantities are plotted, then use the same variable names as in F,G? Colours not explained - presumably contour lines.

We added explicit labels (F1, F2, G1, G2). We only did the simulation of two oral groove positions for reorientation vs. amplitude, to highlight the sensitivity to oral groove position; it did not seem very informative to do it again on panel G.

The oral groove faces the axis of the spiral movement, we inserted a note in the caption.

Reorientation angle is calculated as "the change in angle before and after the stimulus, averaged over 1 second (the spinning period)" (Methods, Behavioral scenarios). We edited panel H to show the same variable names.

We inserted the explanation of the colors (indeed contour lines).

Reviewer #3

I enjoyed reading this paper and I think it is quite suitable for PLoS Comp bio. The authors have done a great deal of physiology and modeling to capture the biophysics of the paramecium action potential. They have made a hydrodynamic model in order to guide the creation of a kinematic description of the movement. They also built a clever closed loop version of the model that uses a simple shape to estimate the size of the stimulus (through the area stimulated). I have a few minor comments.

What or where is the oral groove?

The oral groove is the "mouth" of Paramecium, a sort of channel covered with powerful cilia. We inserted an introductory figure (Fig. 1) showing pictures of Paramecium (Fig. 1A), where the oral groove can be seen.

If I understand it the kinematic model does not model the actual cilia movement but rather uses the calcium concentration in a space clamped model along with the three curve's forms v,theta, omega to drive the paramecium

Yes, this is correct. It is assumed that calcium concentration is the same in all cilia and that it controls ciliary movement, so that all kinematic parameters must depend on calcium concentration.

What accounts for the noise in the trajectories? Is this deterministic chaos, or is there noise added? Or is this just from the spinning? So it's sort of an independent periodic behavior (spinning) decoupled from the linear motion except through the heuristic transfer functions in fig 7? In real cells is there any correlation between spinning and forward movement?

There is some membrane noise in the model in Fig. 11, which accounts for spontaneous action potentials (l. 728, "we added a noisy current to the membrane equation"), but the apparent randomness of the directional changes comes indeed from the spinning, which is periodic and decoupled from the linear motion. In the model, calcium concentration controls spinning speed (omega in Fig. 9C), but the phase is the integrated speed, so it is essentially unrelated to the

history of stimulation (except on short time scales). This is described in real cells by Jennings (see our review, Brette (2021), eNeuro).

Regarding this, it would be interesting to see more details on how the turn is made depending on the phase of the spin (which i guess determines where the oral groove) If I understand it, the oral groove somehow braks the symmetry of the cell and the effects of stimuli are somehow related to this> I may be wrong in which case the authors need to do a better job of explaining how the oral groove alters the motion. So much of the details were buttied in the extremely long methods section.

Yes, the interpretation is correct. The impact of spinning phase in the model is shown in Fig. 9F (red vs. black). What would be particularly interesting would be to measure it in real cells, but unfortunately the oral groove is not clearly visible on our current behavioral data.

On the equation for the calcium dynamics: Are the signs correct in this equation for calcium? I could not find a value for lambda, but it better be negative. I think a minus sign is needed for the calcium influx and if lambda is positive, then it is incorrect

Thank you for spotting this typo! It should be negative indeed. The value is in Table 3, but by mistake we had used the symbol α instead of λ.
